# Deacetylation of Ku70 regulates ionizing-radiation induced DNA damage responses in human cells

**DOI:** 10.1186/1471-2164-15-S2-P24

**Published:** 2014-04-02

**Authors:** Ahmed Al-Emam, Darren Arbon, Boris Kysela

**Affiliations:** 1 DNA Repair and Genome Stability Research Group, School of Clinical and Experimental Medicine, College of Medical and Dental Sciences, University of Birmingham, B15 2TT, UK; 2 Forensic Medicine and Clinical Toxicology Department, Mansoura University, Mansoura, Egypt; 3 Pathology Department, King Khalid University, Abha, KSA

## Background

Ionizing radiation (IR) has unique characteristics as a genotoxic agent in terms of the DNA damage it produces. Its primary lesions, DNA double-strand breaks, are responsible for its biological effects. Ku70 in hetero-trimeric complex with Ku80 and DNA-PKcs represents a crucial component of the non-homologous end joining (NHEJ) DNA double-strand break (DSB) repair machinery. It has been shown previously that the modulation of Ku70 acetylation mediates HDAC inhibitors-induced sensitization of cancer cells to chemotherapy. However, the effect of such modulation on the DNA repair efficacy in response to IR has not been studied in details. Here we investigated the effect of deacetylation of Ku70 lysines K317, K331 and K338 on cellular responses to IR.

## Materials and methods

Site-directed mutagenesis was used to replace Ku70-K317, K331 and K338 with arginine. MRC5VA cells were transfected using lipofectamine and the expression was checked by Western blot and immunoflourescence. For clonogenic survival, irradiated cells were seeded out into 6-well plates along un-irradiated control and cultured for 14 days. The colonies were stained with crystal violet and counted. The cells were grown on coverslips in 6-well dishes, irradiated and stained for γ-H2AX. Slides were analysed with a Carl Zeiss microscope and the foci were counted using Photoshop. The data were statistically analysed using the students’ two-tailed t-test.

## Results

The Clonogenic assay revealed that the survival of the mutant cells is significantly lower than the controls at 8Gy of IR. Moreover, the mutant cells retained statistically significant higher percentages of residual foci 24 hours post irradiation than the controls.

## Conclusions

Our work showed that overexpression of Ku70-K317R, Ku70-K331R and Ku70-K338R mutants sensitizes MRC5VA to IR and impairs their capacity to repair DNA DSBs. This finding strongly implies that the deacetylation of Ku70 plays an important role in the regulation of IR-induced DNA damage responses in human cells and may provide a new route for a therapeutic intervention in cancer treatment.
